# Designing novel possible kinase inhibitor derivatives as therapeutics against *Mycobacterium tuberculosis*: An *in silico* study

**DOI:** 10.1038/s41598-019-40621-7

**Published:** 2019-03-13

**Authors:** Mohd Shahbaaz, Anati Nkaule, Alan Christoffels

**Affiliations:** South African National Bioinformatics Institute (SANBI), SA Medical Research Council Bioinformatics Unit, University of the Western Cape, Private Bag X17, Bellville, 7535 Cape Town South Africa

## Abstract

Rv2984 is one of the polyphosphate kinases present in *Mycobacterium tuberculosis* involved in the catalytic synthesis of inorganic polyphosphate, which plays an essential role in bacterial virulence and drug resistance. Consequently, the structure of Rv2984 was investigated and an 18 membered compound library was designed by altering the scaffolds of computationally identified inhibitors. The virtual screening of these altered inhibitors was performed against Rv2984 and the top three scoring inhibitors were selected, exhibiting the free energy of binding between 8.2–9 kcal mol^−1^ and inhibition constants in the range of 255–866 nM. These selected molecules showed relatively higher binding affinities against Rv2984 compared to the first line drugs Isoniazid and Rifampicin. Furthermore, the docked complexes were further analyzed in explicit water conditions using 100 ns Molecular Dynamics simulations. Through the assessment of obtained trajectories, the interactions between the protein and selected inhibitors including first line drugs were evaluated using MM/PBSA technique. The results validated the higher efficiency of the designed molecules compared to 1^st^ line drugs with total interaction energies observed between −100 kJ mol^−1^ and −1000 kJ mol^−1^. This study will facilitate the process of drug designing against *M*. *tuberculosis* and can be used in the development of potential therapeutics against drug-resistant strains of bacteria.

## Introduction

In the past decades, protein kinases and G protein-coupled receptors have become the most significant group of drug targets for the pharmaceutical industry, with a large number of therapeutic molecules generated through protein kinase based drug optimization programs^1,2^. A majority of designed kinase inhibitors target the ATP binding site of the enzymes^1–3^. In bacteria, the kinases of two-component signal transduction systems involved in the protein phosphorylation are primary used as the drug targets^3^. In recent studies, the roles of bacterial protein kinases in virulence and in the sustainment of growth have been reported^4^. Therefore, bacterial protein kinases can be utilized as potential new drug targets^5^.

In pathogenic bacteria, Polyphosphate kinase - 1 (PPK1), an inner cell membrane-bound enzyme, reversibly catalyze the conversion of terminal inorganic phosphate (Pi) of ATP into the long-chain Polyphosphates^6,7^. This process involves the phosphorylation of histidine residue in the active site of PPK1, followed by the transformation of Pi into Inorganic Polyphosphate (Poly-P) by addition of ATP or back conversion to ATP by the addition of ADP^6,7^. Poly-P is a linear chain polymer of numerous inorganic phosphate residues linked together by phosphoanhydride bond^6,7^. The Poly-P is present ubiquitously in every living cell and plays a variety of physiological functions depending on the sub-cellular localization^6,7^. Poly-P is primarily involved in processes such as substitution for ATP in kinase reactions, chelation of metals, reservoir of Pi, capsule of bacteria, buffer against alkali, mRNA processing, competence for bacterial transformation as well as play regulatory roles in a variety of stress conditions^6,7^. The experimental exposure to the stress conditions lead to the fluctuation in the intracellular level of Poly-P, and decreased concentration is coupled with impairment of various significant structural as well as cellular functionalities^6,7^. Furthermore, in addition to the synthesis of Poly-P, PPK1 also catalyzes the synthesis of nucleoside triphosphates from nucleoside diphosphates by utilizing the Poly-P as phosphate donor^6,7^.

Besides PPK1, another widely conserved family of kinases involved in Poly-P metabolism is known as Polyphosphate kinase 2 (PPK2) enzyme^6,7^. The PPK2 family of enzymes contains a conserved P-loop motif for phosphate binding and is largely categorized into three subfamilies on the basis of substrate specificity (i.e. class I, II and III)^6,7^. The class I as well as class II PPK2 enzymes are involved in the phosphorylation of nucleoside diphosphate and nucleoside monophosphate, whereas, the class III PPK2 enzymes catalyzes the direct synthesis of nucleoside triphosphates from the nucleoside monophosphates^6,7^. However, the exopolyphosphatase (PPX) catalyzes the cleavage of phosphoanhydride bonds of Poly-P and enable the generation of inorganic phosphate^6,7^. The aforementioned enzymes are encoded in the genome of *Mycobacterium tuberculosis* and are involved in both Poly-P synthesis (Rv2984, PPK1) and its utilization (Rv3232c, PPK-2, and Rv0496, PPX). *In vitro* studies in the oxidative and antibiotic stress conditions revealed the accumulation of Poly-P in mycobacteria at a later stage of growth^8^. Furthermore, several studies revealed that the impaired survival of *M*. *tuberculosis* in macrophages is associated with dysregulation in Poly-P levels^8^.

In this study, a library of 18 inhibitors was designed by altering the scaffolds of computationally identified inhibitors using the concepts of combinatorial chemistry and the top three inhibitors were filtered using the virtual screening against Rv2984. Furthermore, the generated docked complexes were further analyzed using 100 ns Molecular Dynamics (MD) simulations in explicit solvent conditions and their conformational behaviors were analyzed, which validated the outcomes of molecular docking. This combined *in silico* study provide significant structural insights into the inhibition of Rv2984 and may enable the identification of potential therapeutic agents against the infection of *M*. *tuberculosis*.

## Results and Discussion

The past decades have seen a resurgence in the research of TB drug design and development, stimulated by an urgent need to curb the rise of the disease globally as well as to develop new, more effective therapies against drug-sensitive and resistant strains^9^. Consequently, there is now a variety of new products in clinical development and historically largest number of early-stage projects aimed at the development of pipelines regarding the formulation of novel compounds were undertaken^9^. It is evident that the integration of the computational approaches with the experimental workflows can accelerate TB drug discovery^10^.

Previous studies used genome and metabolic pathway mapping to identify nine putative targets for the design of novel therapeutic agents^11^. In this study, Rv2984 was used as a target for the design of potential inhibitors against *M*. *tuberculosis*, because of its polyphosphate kinase 1 activity and experimentally confirmed role of this class of protein in virulence mechanisms of bacteria^7^. Accordingly, a library of 18 possible inhibitor molecules was designed (Table S1) using the principles of computational combinatorial chemistry^12^. The generated compounds were used to perform the virtual screening in the predicted binding sites of Rv2984 using GLIDE^13,14^. Therefore, the current study was undertaken to analyze the structural basis of inhibition using the top three scoring ligand molecules in the designed library.

### Structure prediction and assessments

The conservation of Rv2984 was assessed in the genus mycobacteria using multiple sequence alignment performed by PRALINE^15^. Rv2984 showed very high similarity and conservation to the active site histidine residues His435 and His592 to the other mycobacterial polyphosphate kinases (PPKs) and the consensus protein sequence was derived from the alignment (Fig. S1). Consequently, the homology modelling algorithm implemented in “PRIME” was used for the prediction of the Rv2984 structure. BLASTP was used to identify a reliable template in the biological database. Rv2984 showed similarities with the crystal structure of polyphosphate kinases of *Escherichia coli* (PDB ID - IXDO, 34% identity) and *Porphyromonas Gingivalis* (PDB ID - 2O8R, 35% identity). These two templates were selected for building the homology models by satisfying the spatial restraints. The predicted structures were subjected to energy minimization and their loops were further refined using the structure optimization modules present in the Schrödinger software suite. Moreover, the structure of Rv2984 was further refined using 10 ns MD simulations. The model was subjected to subsequent minimization, equilibration and production stages using GROMACS simulation package^16^ in explicit water conditions. The quality of the predicted structure was evaluated using ProQ^17^ and ProSA-web^18^ servers. The ProQ assessed the quality of the predicted model on the basis of LGscore and MaxSub scores, which were calculated to be around 5.565 and 0.534 respectively, classifying the model in extremely good prediction category. Similarly, the ProSA-web calculated the overall quality of Rv2984 model in the form of Z-score of −11.62 indicating the reliable prediction of the 3-D structure. In addition, the 3-D model showed 95.9% of the residues in the allowed region of the Ramachandran plots, demonstrating the stereochemical reliability of the predicted models.

**Figure 1:**
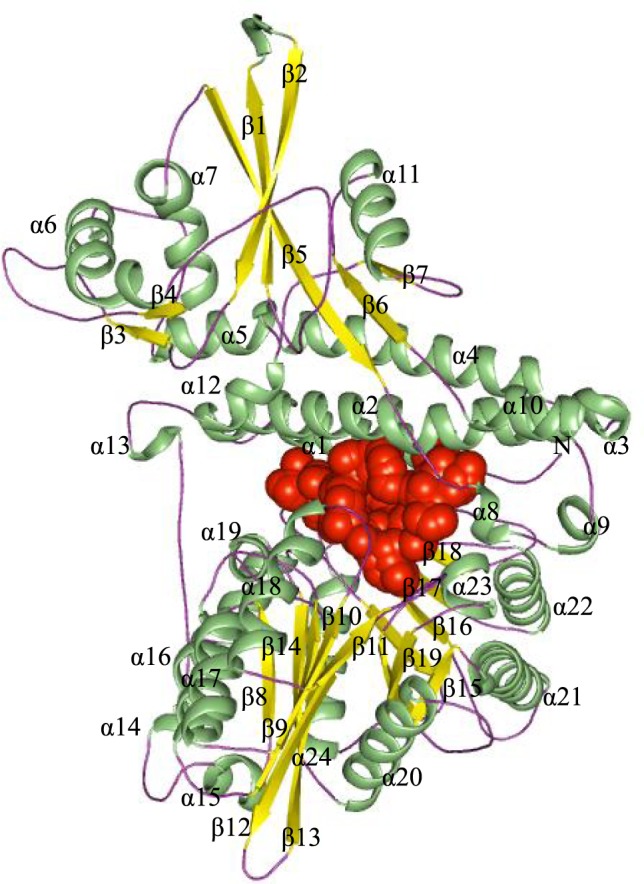
The predicted structure of Rv2984 showing characteristic L-shaped topology organized in 25 α-helices and 19 β-strands. (Spheres depict the predicted binding pocket).

The structural comparison of the predicted Rv2984 structure showed the presence of L-shaped topology with 25 α-helices and 19 β-strands, organized into four structural domains (Fig. 1). After comparison with the template, these four domains were classified as the N-terminal domain, the ‘head’ domain as well as two closely related C-terminal domains^19^. The N-terminal domain, from residues 1–155, comprises three anti-parallel helices (α1–α3). In bacterial PPKs, the N-terminal domain is highly conserved which provides an upper binding interface for the adenine ring of the ATP^19^. The Head domain (Residues 156–375), showed a core α/β/α fold in the middle of the Rv2984 topology and may be involved in the dimerization^19^. The C1 (Residues 376–556) and C2 (557–742) terminal domains contain mixed β-sheet flanked by α-helices. These C-terminal domains are highly conserved and may be associated with the catalytic activity^19^. Afterwards, the pharmacophore models were generated using PHASE pharmacophore generator present in Schrödinger software suite^20^. Receptor-ligand common feature pharmacophoremodels were generated and the drugs bearing the same features were searched in the available databases. Consequently, the 18 compound derivatives were designed using combinatorial approaches (Table S1). The drug-likeness of the designed molecules were assessed using “Ligand-based ADME/Tox Prediction” Module and validated using vNN server for ADMET Predictions^21^. The molecules showed the presence of no cytotoxicity, low metabolism in the liver, no cardiotoxicity, no mitochondrial toxicity as well as no mutagenicity. Therefore, these molecules were selected for further study.

### Docking-based interaction studies

The analyses of the active site of template PPK (PDB ID – IXDO) showed that His435 and His592 function as the autophosphorylation sites^19^. After structural analyses, the corresponding autophosphorylation sites were located in the residues His491 and His652 of predicted Rv2984 structure. The Histidine residue is highly conserved in the core active sites of kinases and usually forms the part of the structural motifs such as His-x-Asp^22^. The replacement of these His residues in His-x-Asp motifs with Phenylalanine or Arginine leads to the impairment in the catalytic as well as autophosphorylation activity^22^. The probable active site pockets were predicted using the AutoLigand module of the AutoDock software suite^23^. The hotspot sites for the drug designing were further validated in the structure of Rv2984 using Fpocket^24^ and 3DLigandSite^25^ servers, which identified 72 ligand binding sites and recognizes the ATP in the binding site. The pocket 1 (Depicted in Fig. 1) was selected for the virtual screening. Subsequently, the designed derivative molecules were docked in the predicted active site of Rv2984. The abbreviated names of the used derivatives were listed in Table S1.

**Figure 2:**
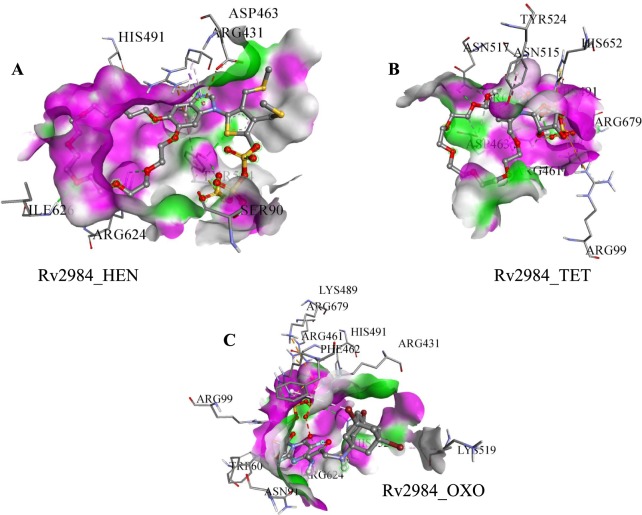
The energy minimized conformations showing (**A)** The 36^th^ docked pose for the Rv2984_HEN complex, (**B)** Selected 30^th^ pose of complex Rv2984_TET, and (**C)** The generated stable pose of the Rv2984_OXO complex.

The docked pose 36 was selected for the complex Rv2984_HEN (Table 1) and it was observed that HEN was interacting with Ser90, Arg431, Asp463, His491, Tyr524, Arg624, and Ile626 (Fig. 2A). The HEN derivative showed the highest value of the inhibition constant of 866.29 nM along with the free energy of binding calculated to be around −8.27 kcal mol^−1^ (Table 1). Similarly, the TET was found to be interacting with Arg99, Arg431, Arg461, Asp463, His491, Asn515, Asn517, Tyr524, His652, and Arg679 (Fig. 2B) in the selected docked pose 30. In complex Rv2984_TET, the least value of inhibition constant of 255.33 nM was calculated, while it showed the highest free energy of binding of −8.99 kcal mol^−1^ (Table 1). Furthermore, in Rv2984_OXO complex, the pose 2 was selected and the residues Trp60, Asn91, Arg99, Arg431, Arg461, Phe462, Lys489, His491, Lys519, Arg624, His652, and Arg679 were found to be interacting with OXO (Fig. 2C). The respective complex showed the combination of the free energy of binding and inhibition constant to be around −8.56 kcal mol^−1^ and 528.83 nM respectively. Both the potential autophosphorylation sites i.e. His491 and His652 are observed in the Rv2984_TET and Rv2984_OXO complexes, while only His491 was observed in Rv2984_HEN (Fig. 2), indicating the binding efficiency of TET and OXO derivatives with Rv2984.

**Table 1:** List of generated docking scores and parameters for Rv2984.

S. No	Complex name	Selected Docked Pose	Free energy of Binding (kcal/mol)	Inhibition constant (nM)	Ligand Efficiency	vdW + Hbond + desolv Energy (kcal/mol)	Intermolecular energy (kcal/mol)	Total internal (kcal/mol)	Torsional energy (kcal/mol)
1.	Rv2984_HEN (RMS = 1.42)	36	−8.27	866.29	−0.15	−12.02	−11.85	−3.02	3.58
2.	Rv2984_TET (RMS = 0.2)	30	−8.99	255.33	−0.21	−8.33	−11.38	−5.79	2.39
3.	Rv2984_OXO (RMS = 0)	2	−8.56	528.83	−0.17	−10.47	−13.34	−4.43	4.77
4.	Rv2984_ISO (RMS = 0.64)	19	−4.64	393.73	−0.46	−4.87	−5.24	−0.51	0.6
5.	Rv2984_RIF (RMS = 0.74)	33	−9.74	72.83	−0.17	−11.36	−11.53	−0.19	1.79

**Figure 3:**
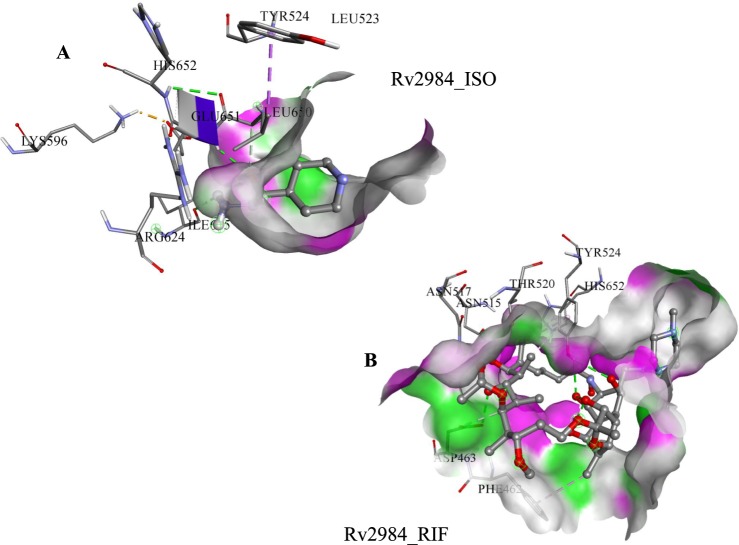
Docking results of 1st line drugs. (**A**) The selected conformational pose of Rv2984_ISO docked complex illustrating the interacting residues and (**B)** The energetically stabilized complex of first-line drug RIF.

Experimental studies have identified that the PPK is associated with the reduction of first-line drugs susceptibility as well as required for intracellular survival of *M*. *tuberculosis* in macrophages^8,26,27^. Therefore, the first line drugs such as Rifampicin (RIF) and Isoniazid (ISO) was docked in the active pocket of Rv2984 and their binding affinities were compared with the designed derivatives. In Rv2984_ISO complex, the free energy of binding of −4.64 kcal mol^−1^ and inhibition constant of 393.73 nM was observed (Table 1). The selected 19th docked pose of Rv2984_ISO complex showed interaction with Leu523, Tyr524, Lys596, Arg624, Ile645, Leu650, Glu651, and His652 (Fig. 3A). Similarly, in Rv2984_RIF complex the drug showed the free energy of binding and inhibition constant of −9.74 kcal mol^−1^ and 72.83 nM respectively, with Phe462, Asp463, Asn515, Asn517, Thr529, His652, and Tyr524 were observed in interaction pocket (Fig. 3B). In first line drug complexes, only His652 was observed in the interaction site, but the structural comparison with template showed that the primary autophosphorylation sites may be located at His491.

Therefore, the designed inhibitor derivates probably target the autophosphorylation sites in the Rv2984 with comparable efficiency in comparison with the first line drugs. These generated parameters showed that designed derivatives are favorably binding in the active sites of Rv2984 and can be used to inhibit the function of the protein. These observations emphasized the suitability of these designed inhibitors to be used as potential therapeutics against *M*. *tuberculosis*. Furthermore, in order to obtain deeper insight into the generated complexes, each of them was subjected to 100 ns MD simulations in explicit water conditions and correlation of these observations with information existing in the literature are discussed in the following section.

### Changes in molecular dynamics patterns

In addition to the molecular docking studies, the Molecular Dynamics (MD) simulations were used to obtain a deeper insight into the structural changes observed during the course of inhibition. The MD simulations were widely used in the process of structure-based drug designing, for generating the structural conformer of the receptor crystal structure before docking as well as for predicting the most energetically minimized binding modes of the top ranking lead compounds as a final filter for guiding the chemical synthesis for hit optimization^28^. Consequently, all the generated docked complexes were immersed in the SPC/E water model and then subjected to the process of minimization for 1000 steps of steepest descent, followed by equilibration and finally subjected to 100 ns MD simulations. The trajectories generated for each complex were analyzed using the utilities present in GROMACS and the obtained outcomes were discussed in the following sections of the article.

#### Variations in bonding patterns demonstrate that the combinatorial designed inhibitors bind more tightly than the 1st line anti-TB drugs

**Figure 4:**
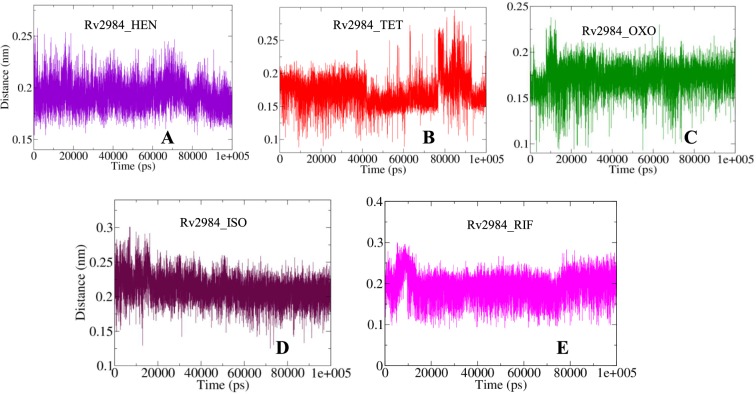
The graphical illustration of changes observed in the distances between Rv2984 and the complexed ligand molecules (**A–E**) during the course of 100 ns MD simulations.

The gmx_mpi pairdist module was used to calculate the distance between the structure of Rv2984 and the ligand molecules during the course of simulations. The average distance for the complex Rv2984_HEN was observed to be around 0.19 nm (Fig. 4A); while for the complexes Rv2984_TET and Rv2984_OXO it was computed to be around 0.17 nm and 0.18 respectively (Fig. 4B,C). Likewise, an average distance of 0.21 nm was observed for the complex Rv2984_ISO as well as for the Rv2984_RIF average value of 0.20 nm was computed (Fig. 4D,E).

**Figure 5:**
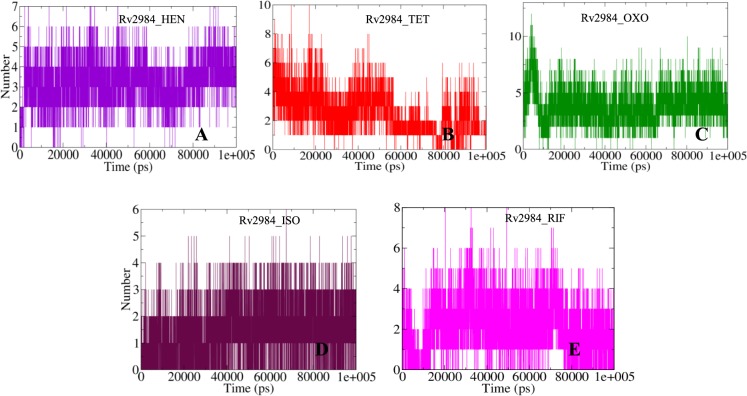
The 2-D diagram describing the dynamics observed in the hydrogen bonding patterns for the studied Rv2984 complexes (**A–E**) for the time scale of 100 ns.

Furthermore, the bonding patterns were further assessed by observing the fluctuations of the hydrogen bonds in the studied complexes (Fig. 5). Around seven hydrogen bonds were observed in complex Rv2984_HEN (Fig. 5A), while in complexes Rv2984_TET and Rv2984_OXO around 10 and 12 hydrogen bonds were computed respectively (Fig. 5B,C). Similarly, around six hydrogen bonds were calculated for the complex Rv2984_ISO and eight for Rv2984_RIF (Fig. 5D,E). These observations indicated that the OXO showed highest bonding parameters followed by TET and HEN, which were binding more tightly than the first line drugs.

#### Assessment of structural compactness

**Figure 6:**
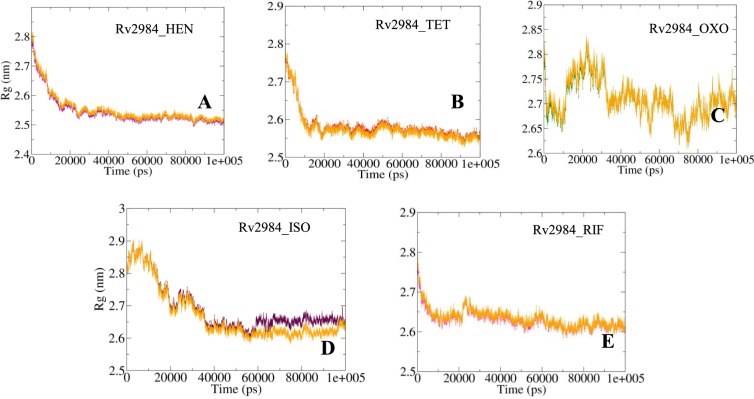
Radius of Gyration plots highlighting the changes observed in the conformational behavior of the studied docked complexes (**A–E**) during 100 ns MD simulations. (The changes in the Rg values for the free protein is depicted in orange color).

The Radius of gyration (*R*_*g*_) is a parameter used to evaluate the stability of the complexed biological systems along the MD trajectories by calculating the structural compactness of the biomolecules. The average *R*_*g*_ values for complexes Rv2984_HEN and Rv2984_TET were calculated to be around 2.54 nm and 2.58 nm respectively, in which the systems become relatively stable as the MD simulations approach towards 100 ns time scale (Fig. 6A,B). While for complex Rv2984_OXO highest number of fluctuations was observed throughout the course of simulations with average *R*_*g*_ value of 2.70 nm (Fig. 6C). Furthermore, the average *R*_*g*_ value for complexes Rv2984_ISO and Rv2984_RIF was computed to be around 2.68 nm and 2.63 nm respectively, with lesser fluctuation during 100 ns MD simulations (Fig. 6D,E). These findings indicated that the highest structural compactness was observed in the complexes Rv2984_HEN and Rv2984_TET, while higher fluctuations were observed in Rv2984_OXO which attributed to the active interaction of Rv2984 with the ligand. Furthermore, in comparison with free protein structure, the relatively similar behavior of compactness was observed for each complex (Fig. 6).

#### Deviations in conformational elements

**Figure 7:**
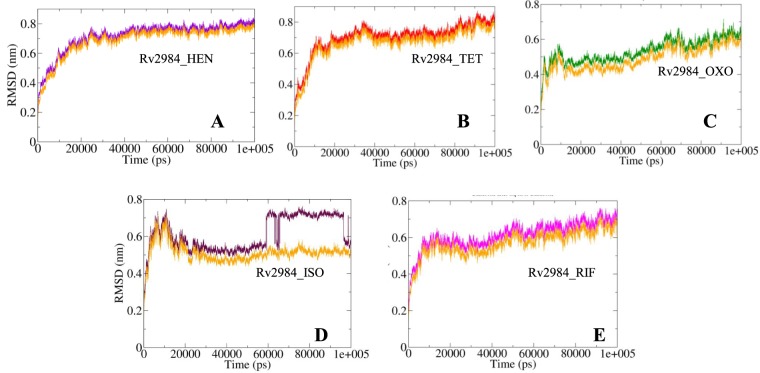
The curves illustrating the changes in the RMSD values for each studied complex (**A–E**) during 100 ns MD simulations. (The changes in the RMS values for the free protein is depicted in orange color).

The evaluations of structural similarities are considered as a crucial method for the prediction of protein functions as well as their folding in 3-D space. Therefore, the differences in the conformations of the structural elements were compared in order to obtain the stability profiles of the studied complexes. The Root Mean Square Deviation (RMSD) is a parameter aimed at the calculation of the deviations in the conformational stability of macromolecules from the backbone structure to the initial starting structure. The complexes Rv2984_HEN and Rv2984_TET showed a similar pattern of RMSD values fluctuations, with average RMSD values of 0.71 nm and 0.70 nm respectively (Fig. 7A,B). While for complex Rv2984_OXO, the average value was calculated to be around 0.53 nm with fluctuation observed in the range of 0.4–0.6 nm (Fig. 7C). This indicated that the complex Rv2984_OXO is relatively stable in comparison to the other designed derivatives’ complexes. In comparison with the complexes of the first line drugs, the Rv2984_ISO showed the average value of 0.61 nm with relatively higher perturbations indicating the unstable nature of the complex (Fig. 7D). Similarly, the Rv2984_RIF showed 0.62 nm as the average RMSD value with the inclined fluctuation pattern (Fig. 7E). These observations indicated that the Rv2984_OXO showed a relatively stable RMSD profile in comparison to the other complexes, while free proteins showed little changes in the RMSD values while compared with the complexed form (Fig. 7).

#### Fluctuations in the residual components

**Figure 8:**
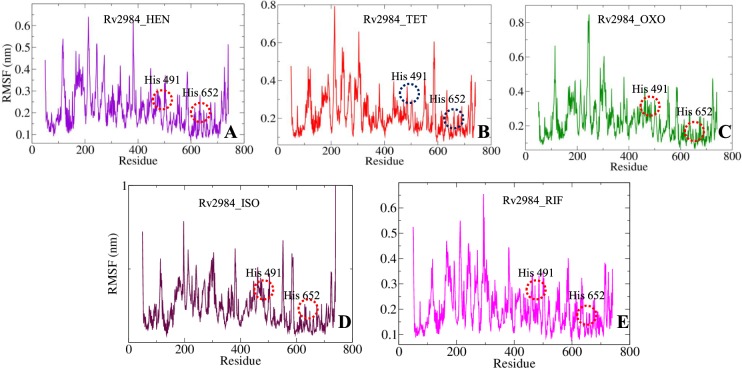
The graphs highlighting the changes observed in the fluctuations of the constituent residues involved in the interaction of the Rv2984 with the ligand molecules in the studied complexed systems (**A–E**).

The variations in the constituent residues of Rv2984 were observed for each complex and were plotted as a function of time during the course of 100 ns MD simulations (Fig. 8). The His491 and His652 form the part of loop regions adjacent to β12 and β17 (Fig. 1). In complex Rv2984_HEN, the His491 and His652 showed Root Mean Square Fluctuations (RMSF) of 0.151 nm and 0.136 nm respectively (Fig. 8A). The complex Rv2984_TET showed relative active interactions with the residues as indicated by the RMSF values of 0.192 nm and 0.114 nm (Fig. 8B). While in complex Rv2984_OXO the least RMSF values of 0.152 nm and 0.101 nm were observed for the respective residues (Fig. 8C). Furthermore, in complex Rv2984_ISO the residues showed active interaction with the first line drug isoniazid which can be indicated from the RMSF values of 0.207 nm and 0.125 nm (Fig. 8D). Moreover, in Rv2984_RIF the residues showed RMSF values of 0.182 nm and 0.128 nm, which slightly lesser as compared to isoniazid complex (Fig. 8E). These plotted data showed that the complexes of designed derivatives showed active interaction with His491 and His652 and comparable RMSF values with 1st line TB drugs.

#### Alterations in solvent accessibility

**Figure 9:**
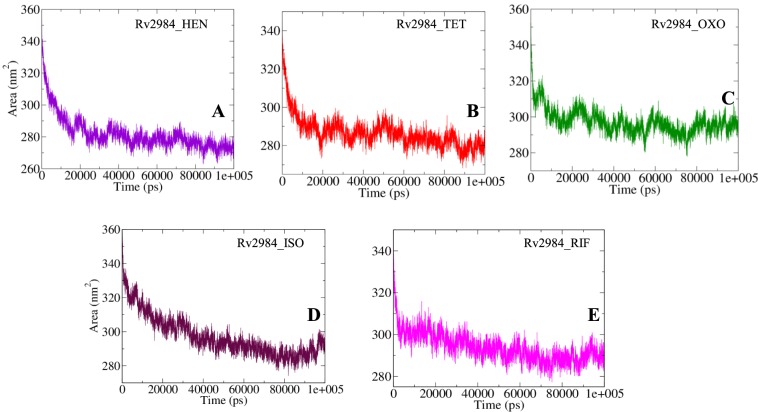
The SASA curves showing the variation in the solvent accessibility of the studied complexes (**A–E**) during the course of 100 ns MD simulations.

The Solvent Accessible Surface Area (SASA) is a parameter computed using gmx_mpi sasa module of GROMACS, which measures the proportion of the biomolecules surface interacting with the water solvent. The calculation of SASA can be used for predicting the extent of the conformational changes occurred during the course of binding^29^. The average SASA of 281.97 nm^2^ was calculated for Rv2984_HEN, while for Rv2984_TET and Rv2984_OXO complexes it was found to be around 286.63 nm^2^ and 297.22 nm^2^ respectively (Fig. 8A–C). Likewise, the Rv2984_ISO and Rv2984_RIF showed the average value of SASA to be around 297.25 nm^2^ and 293.87 nm^2^ (Fig. 9D,E). These calculations showed that the Rv2984_HEN was least exposed to the water solvent during 100 ns MD simulations followed by Rv2984_TET, indicated the relatively stable nature of these complexes as compared to the rest of the studied systems.

#### Dynamics of interaction energies

**Figure 10:**
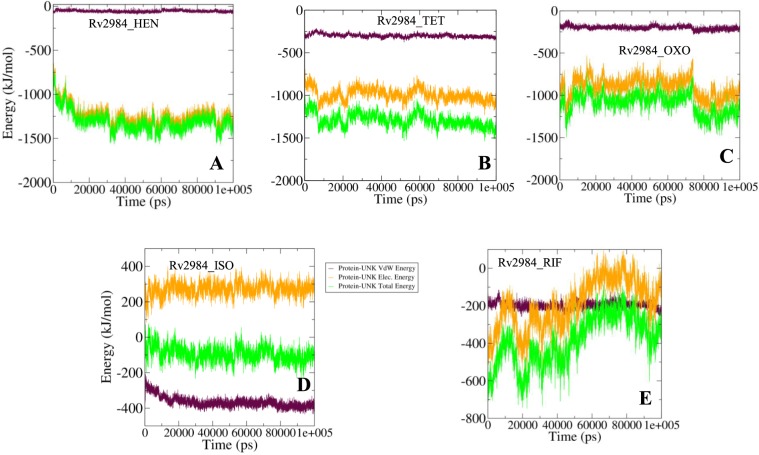
The MM-PBSA analysis of docked complexes based resulted curves (**A–E**) showing the behavior of the interactions in the form of free energies of binding between the Rv2984 and ligand molecules.

In order to validate the binding energy parameters generated using molecular docking studies, a detailed analysis was performed regarding the calculation of the free energies of interaction associated with the binding of ligand molecules with the structure of Rv2984 using MM–PBSA method implemented in GROMACS. The total binding energies of all the designed derivative molecules and Rv2984 were observed in the range of −1000–1500 kJ mol^−1^ (Fig. 10A–C). While for the complexes generated using first-line drugs the total energy was calculated to be around −200 kJ mol^−1^ (Fig. 10D,E). These interaction energy calculations validated the molecular docking results, showing that the designed derivative molecules were favorably binding to the Rv2984 and can use as potential drug molecules.

## Methods

### Molecular modelling

Due to the unavailability of Three Dimensional (3-D) structures for Rv2984 (UniProt ID - P9WHV9) in biological databases, its structure was predicted using “PRIME” homology modelling module of Schrödinger^30,31^. The generated 3-D structures of Rv2984 were subjected to further optimization which includes minimization, side chain as well as loop refinement. The structures of designed ligands were constructed by means of drawing utilities present in the MAESTRO (Schrödinger Release 2018-1: Maestro, Schrödinger, LLC, New York, NY, 2018) and the geometries of the resulted ligand structures were optimized by using the Density Functional Theory (DFT) method present in JAGUAR^32^.

### Molecular docking studies

The obtained structures generated ligands were docked in the potential binding sites of Rv2984 by using AutoDock 4^33^ package. This software performs the prediction of the bound conformation based on the free energy, which was calculated on the basis of the empirical force field and the Lamarckian Genetic Algorithm^33^. The AutoGrid module was used to create a grid box of dimensions 50 × 54 × 46 Å along the XYZ directions with a spacing of 0.375 Å. The efficiency of the predictions was amplified by setting the parameters associated with the Lamarckian genetic algorithm to the maximum efficiency values. This was achieved by setting number of individuals in population to 250 and the maximum number of energy evaluations to a “longer” interval. The 100 docked conformations were generated for each Rv2984 and ligand systems which were further grouped according to the RMSD tolerance of 2.0 Å. The free energy of binding was used as a parameter for assessing the efficiency of interactions, was calculated on the basis of the given equation^33^:

$$\begin{array}{c}{\rm{\Delta }}{\rm{G}}={{\rm{\Delta }}{\rm{G}}}_{{\rm{vdW}}}\sum _{{\rm{i}},{\rm{j}}}(\frac{{{\rm{A}}}_{{\rm{ij}}}}{{{\rm{r}}}_{{\rm{ij}}}^{12}}-\frac{{{\rm{B}}}_{{\rm{ij}}}}{{{\rm{r}}}_{{\rm{ij}}}^{6}})+{{\rm{\Delta }}{\rm{G}}}_{{\rm{Hbond}}}\sum _{{\rm{i}},{\rm{j}}}{\rm{E}}({\rm{t}})(\frac{{{\rm{C}}}_{{\rm{ij}}}}{{{\rm{r}}}_{{\rm{ij}}}^{12}}-\frac{{{\rm{D}}}_{{\rm{ij}}}}{{{\rm{r}}}_{{\rm{ij}}}^{10}}+{{\rm{E}}}_{{\rm{Hbond}}})\\ \,\,\,+{{\rm{\Delta }}{\rm{G}}}_{{\rm{elec}}}\sum _{{\rm{i}},{\rm{j}}}\frac{{{\rm{q}}}_{{\rm{i}}}{{\rm{q}}}_{{\rm{j}}}}{{\rm{\varepsilon }}({{\rm{r}}}_{{\rm{ij}}}){{\rm{r}}}_{{\rm{ij}}}}+{{\rm{\Delta }}{\rm{G}}}_{{\rm{tor}}}{{\rm{N}}}_{{\rm{tor}}}+{{\rm{\Delta }}{\rm{G}}}_{{\rm{sol}}}\sum _{{\rm{i}},{\rm{j}}}{{\rm{S}}}_{{\rm{i}}}{{\rm{V}}}_{{\rm{j}}}{{\rm{e}}}^{(-{{\rm{r}}}_{{\rm{ij}}}^{2}/2{{\rm{\sigma }}}^{2})}\end{array}$$

here, ΔG_vdW_ symbolizes the van der Waals, ΔG_Hbond_ represents Hydrogen Bonding while ΔG_elec_ and ΔG_sol_ stand for the free energy of Electrostatics and solvation, respectively.

The re-scoring of the generated conformations were performed on the basis of the scoring function present in DrugScoreX server^34^ and the docked conformation with the highest score was selected for the Molecular Dynamics (MD) simulations. The generated outcomes were validated using the Glide docking module implemented in Schrödinger^13^.

### Molecular dynamics (MD) simulations

The obtained docked complexes were subjected to MD simulations using GROMACS 5.1.2^16,35^ and the topologies of the complexed structures were generated using GROMOS96 53a6 force field^36^. The GROMACS package lack an appropriate force field parameters for drug-like molecules, therefore, the PRODRG server^37^ was used for the generation of topologies and coordinate files of the ligand molecules. Furthermore, the partial charges in the generated topologies were corrected using DFT method implemented in GAUSSIAN^38^ which utilized the B3LYP 6–31 G (d,p) basis set and the CHELPG program^39^. Afterward, all the docked complexes were solvated in SPC/E water model^40^ as well as the neutralization was performed by adding the counter ions. Consequently, the neutralized systems were energetically minimized by steepest descent and conjugate gradient algorithms with a convergence criterion of 0.005 kcal/mol and the restraints were applied to the structure of the ligands before the equilibration phase.

The equilibration step was performed in NVT (constant volume) as well as NPT (constant pressure) ensemble conditions, each with a 100 ps time scale. The temperature of the system was maintained at 300 K using Berendsen weak coupling method in both ensemble conditions as well as the pressure, which was maintained at 1 bar by utilizing Parrinello-Rahman barostat in constant pressure ensemble. The final MD simulations were produced using LINCS algorithm for 100 ns time scale. The generated trajectories were used to analyze the behavior of each complex in the explicit water environment. The deviations in the distances, H-bonds, RMSD (Root Mean Square Deviations), and Radius of Gyration (Rg) were analyzed between the complexed protein and ligand. Furthermore, the free energy of binding was calculated using Molecular mechanics Poisson–Boltzmann surface area (MM-PBSA) protocols implemented in the *g_mmpbsa* package^41^ which provide deeper insights into the interaction mechanisms of the proteins and the ligand molecules.

## Conclusions

This study involves the design of novel inhibitor molecules against the Rv2984 protein which functions as polyphosphate kinase in *Mycobacterium tuberculosis*. Consequently, a library of 18 derivative molecules was designed using a variety of *in silico* techniques. The interaction patterns of the selected designed molecules were analyzed using molecular docking techniques and the generated parameters were compared with that obtained for the first line drugs demonstrates that the designed molecules bind more efficiently to Rv2984. These detailed computational analyses provide deeper structural insight into the interacting residues of Rv2984 involved in the process of inhibition. Furthermore, the MD simulation studies were used in order to understand the conformational behaviors of the studied Rv2984 complexes with the ligand molecules. The outcomes of MM-PBSA technique validated the molecular docking studies and showed that the designed derivative molecules were binding efficiently to the structure of Rv2984. These detailed analyses indicated that the binding properties of the designed molecules are comparable to the first line drugs and can be utilized as potential therapeutic agents against *M*. *tuberculosis* infection.

## Supplementary information

Table S1 and Figure S1
