# Supplementary material for: Designing novel possible kinase inhibitor derivatives as therapeutics against Mycobacterium tuberculosis: An in silico study
Source: Sci Rep. 2019 Mar 13;9:4405. doi: 10.1038/s41598-019-40621-7 (PMC6416319; doi:10.1038/s41598-019-40621-7)

**Designing novel possible kinase inhibitor derivatives as therapeutics against *Mycobacterium tuberculosis*: An *in silico* study**

Mohd Shahbaaz, Anati Nkaule and Alan Christoffels^*^

*South African National Bioinformatics Institute (SANBI), SA Medical Research Council*

*Bioinformatics Unit, University of the Western Cape, Private Bag X17, Bellville 7535, Cape Town, South Africa*

| S. No  **Table S1**: List of the designed inhibitors against polyphosphate kinase – 1 of *M. tuberculosis*. | Compound | IUPAC name | 2-D structure |
| --- | --- | --- | --- |
|  | 001 | (1R)-1-{[(1P,3R,4R)-2,4-bis({[(1S)-1-hydroxyethoxy]methyl})-5-[(26S,30S)-2,5,8,11,14,17,20,23-octaoxa-27,29-diazatricyclo[22.7.0.0²⁶,³⁰]hentriacont-1(24)-en-27-yl]-3,4-dihydro-1λ⁴-thiophen-3-yl]methoxy}ethan-1-ol | 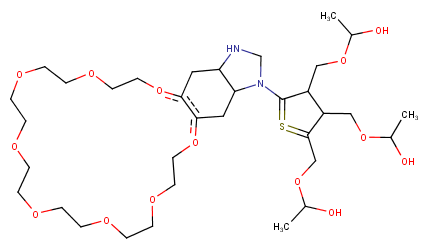 |
|  | 002 | {4-[(ethylamino)(hydroxy)methyl]-5-{2,5,8,11,14,17,20,23-octaoxa-27,29-diazatricyclo[22.7.0.0²⁶,³⁰]hentriacont-1(24)-en-27-yl}-2-({[({[(oxosulfonylidene)-λ⁴-oxidanylidene]sulfonylidene}-λ⁴-oxidanylidene)sulfonylidene]-λ³-oxy}methyl)-3,4-dihydro-1λ⁴-thiophen-3-yl}[(1,2,3,4,8,10-hexahydroadamantan-1-yl)amino]methanol | 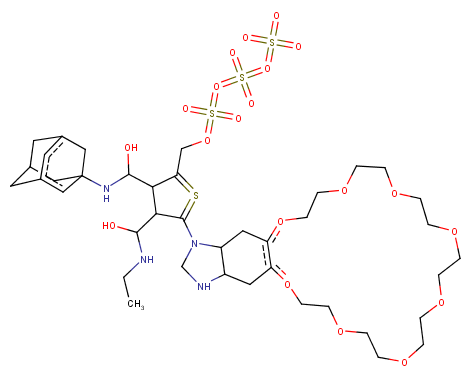 |
|  | 003 | {4-[(ethylamino)(hydroxy)methyl]-5-{34,36,40,41,42,43-hexaazadecacyclo[28.9.1.1³,¹⁰.1¹²,¹⁹.1²¹,²⁸.0⁴,⁹.0¹³,¹⁸.0²²,²⁷.0³¹,³⁹.0³³,³⁷]tritetraconta-1,2,10,11,19,20,28,29-octaen-34-yl}-2-({[({[(oxosulfonylidene)-λ⁴-oxidanylidene]sulfonylidene}-λ⁴-oxidanylidene)sulfonylidene]-λ³-oxy}methyl)-3,4-dihydro-1λ⁴-thiophen-3-yl}[(1,2,3,4,8,10-hexahydroadamantan-1-yl)amino]methanol | 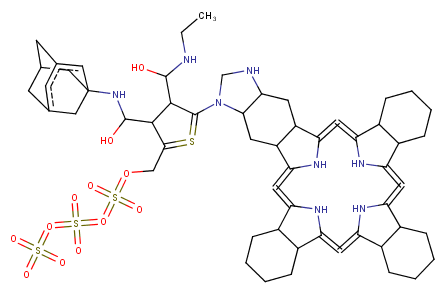 |
|  | 004  (OXO) | 2-[2,5-difluoro-6-hydroxy-4-({2-hydroxy-3-[(3,5,7-tribromoadamantan-1-yl)amino]propyl}amino)-1,3-diazinan-1-yl]-5-({[({[(oxosulfonylidene)-λ⁴-oxidanylidene]sulfonylidene}-λ⁴-oxidanylidene)sulfonylidene]-λ³-oxy}methyl)oxolane-3,4-diol | 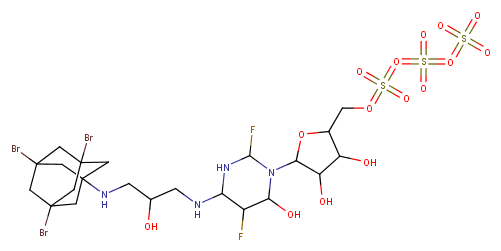 |
|  | 005  (TET) | (1E,10Z,13Z,19Z)-21-[3,4-dihydroxy-5-({[(oxosulfonylidene)-λ⁴-oxidanylidene]sulfonylidene}-λ³-oxy)oxolan-2-yl]-3,6,9,12,15,18-hexaoxa-21,23-diazabicyclo[18.3.1]tetracosa-1,10,13,19-tetraene-2,22-diol | 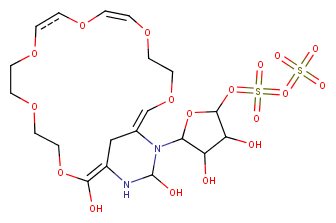 |
|  | 006 | 1,5-dibromo-7-{[2-hydroxy-1-(2-{[hydroxy({2,2,7,7,12,12,17,17-octamethyl-21,22,23,24-tetraazapentacyclo[16.2.1.1³,⁶.1⁸,¹¹.1¹³,¹⁶]tetracosan-4-yl})methyl]amino}ethyl)-1,3-diazinan-4-yl]amino}-4,5,6,7,8,9-hexahydro-1H-adamantan-2-yl | 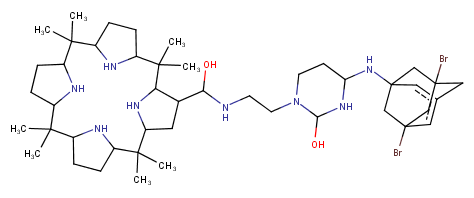 |
|  | 007 | No Name generated | 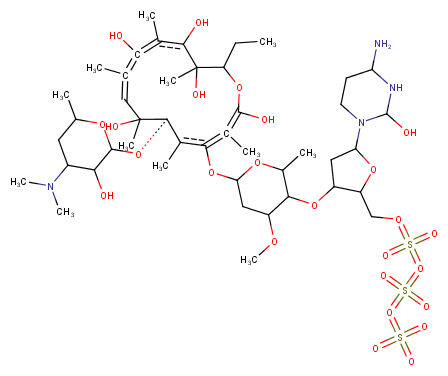 |
|  | 008 | 27-{3,4,5-tris[(methylidene-λ⁴-sulfanylidene)methyl]-3,4-dihydro-1λ⁴-thiophen-2-yl}-2,5,8,11,14,17,20,23-octaoxa-27,29-diazatricyclo[22.7.0.0²⁶,³⁰]hentriacont-1(24)-ene | 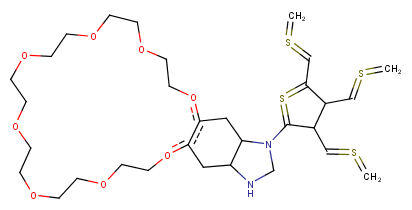 |
|  | 009  (HEN) | 27-{3,4-bis[(methylidene-λ⁴-sulfanylidene)methyl]-5-({[({[(oxosulfonylidene)-λ⁴-oxidanylidene]sulfonylidene}-λ⁴-oxidanylidene)sulfonylidene]-λ³-oxy}methyl)-3,4-dihydro-1λ⁴-thiophen-2-yl}-2,5,8,11,14,17,20,23-octaoxa-27,29-diazatricyclo[22.7.0.0²⁶,³⁰]hentriacont-1(24)-ene | 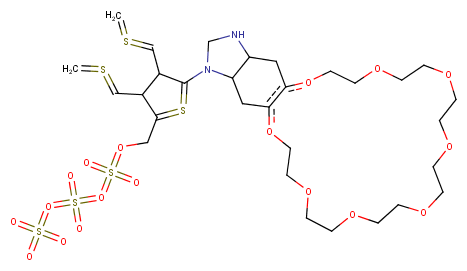 |
|  | 010 | No Name generated | 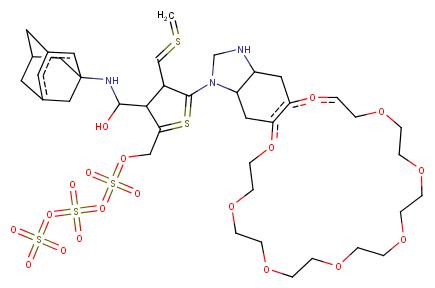 |
|  | 011 | 2-{6-[(3,5,7-tribromoadamantan-1-yl)amino]-octahydro-1H-purin-9-yl}-5-{2-[(3,5,7-tribromoadamantan-1-yl)amino]ethyl}oxolane-3,4-diol | 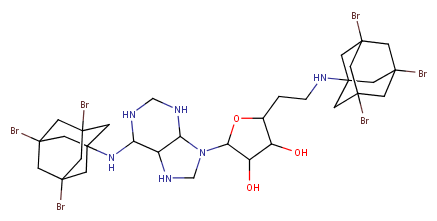 |
|  | 012 | 1,5-dibromo-7-{[3-({9-[3,4-dihydroxy-5-(hydroxymethyl)oxolan-2-yl]-octahydro-1H-purin-6-yl}amino)butyl]amino}-4,5,6,7,8,9-hexahydro-1H-adamantan-2-yl | 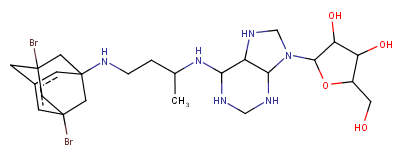 |
|  | 013 | 1,5-dibromo-7-[(5-{4-[(1,2,3,4,8,10-hexahydroadamantan-1-yl)amino]-octahydro-1H-1,3-benzodiazol-1-yl}-3,4-dihydroxyoxolan-2-yl)amino]-4,5,6,7,8,9-hexahydro-1H-adamantan-2-yl | 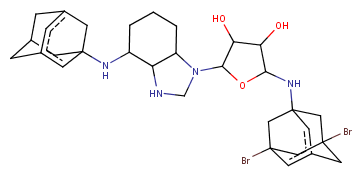 |
|  | 014 | 2-(2-{[(2-chloroethyl)[(2-chloroethyl)-λ³-oxidanylidene]phosphoroso]-λ³-oxy}ethyl)-5-{6-hydroxy-4-[(tetradecahydroacridin-10-yl)methyl]-1,3-diazinan-1-yl}oxolane-3,4-diol | 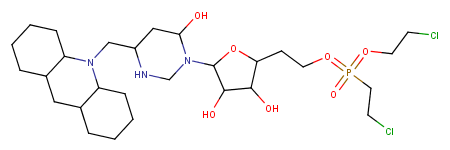 |
|  | 015 | 6-amino-2-({[2-(4-chlorocyclohexyl)-3,4-dihydro-1λ⁴,3-thiazol-4-yl]methylidene}-λ⁴-sulfanylidene)-3-[3-hydroxy-4-(2-hydroxyethoxy)-5-(hydroxymethyl)oxolan-2-yl]-1,3-diazinan-4-ol | 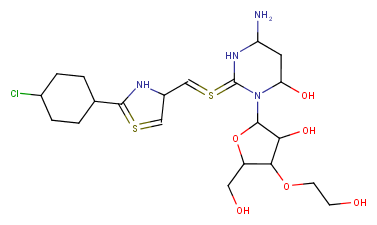 |
|  | 016 | 3-(4-{2-[(6-amino-9-{5-[(ethylidene-λ⁴-sulfanylidene)(hydroxy)methyl]-3,4-bis(1-hydroxyethoxy)oxolan-2-yl}-octahydro-1H-purin-2-yl)amino]ethyl}cyclohexyl)propane-1,1-diol | 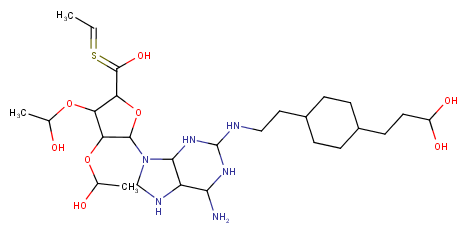 |
|  | 017 | 2-[(4-{[(9-amino-tetradecahydroacridin-3-yl)oxy](hydroxy)methyl}-2-hydroxy-1,3-diazinan-1-yl)methyl]-5-[({[(oxosulfonylidene)-λ⁴-oxidanylidene]sulfonylidene}-λ³-oxy)methyl]oxolane-3,4-diol | 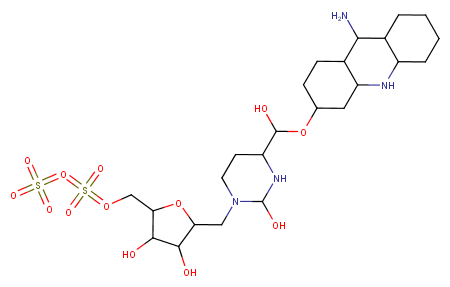 |
|  | 018 | 1-(5-{[({[(9-amino-tetradecahydroacridin-2-yl)-λ³-oxidanylidene]sulfonylidene}-λ⁴-oxidanylidene)sulfonylidene]-λ³-oxy}-3-hydroxyoxolan-2-yl)-4-(hydroxyamino)-1,3-diazinan-2-ol | 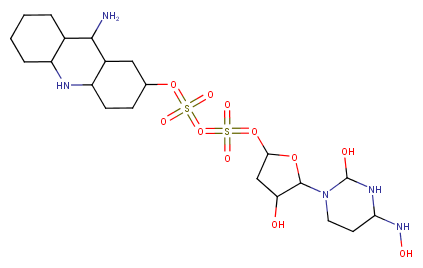 |

Note: Compound 004 abbreviated at “OXO”, compound 005 abbreviated as “TET” and compound 009 abbreviated as “HEN”

**Figure S1**: Depiction of Conservation of Rv2984 (Uniprot ID P9WHV9 ) and Consensus after multiple alignment studies


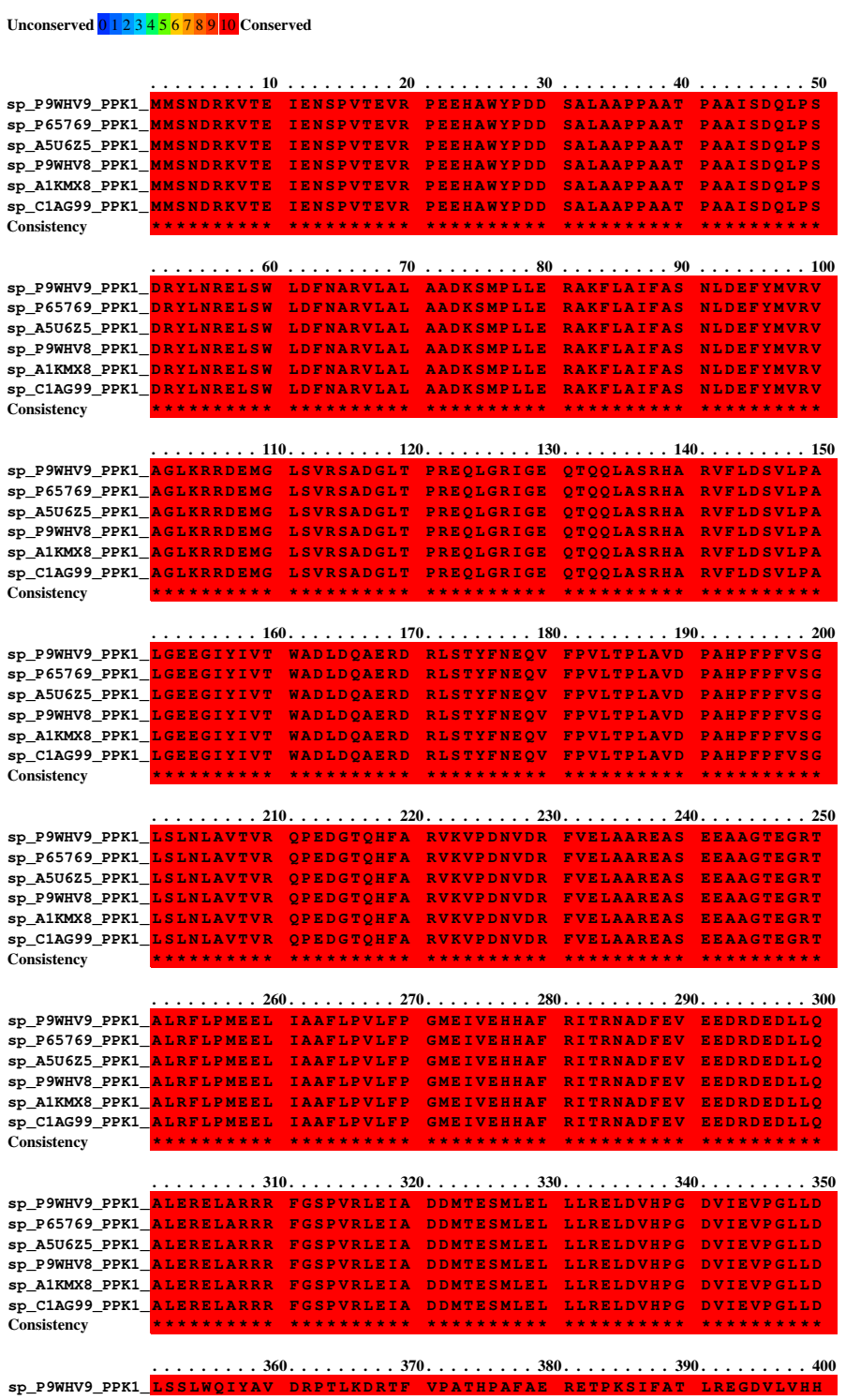


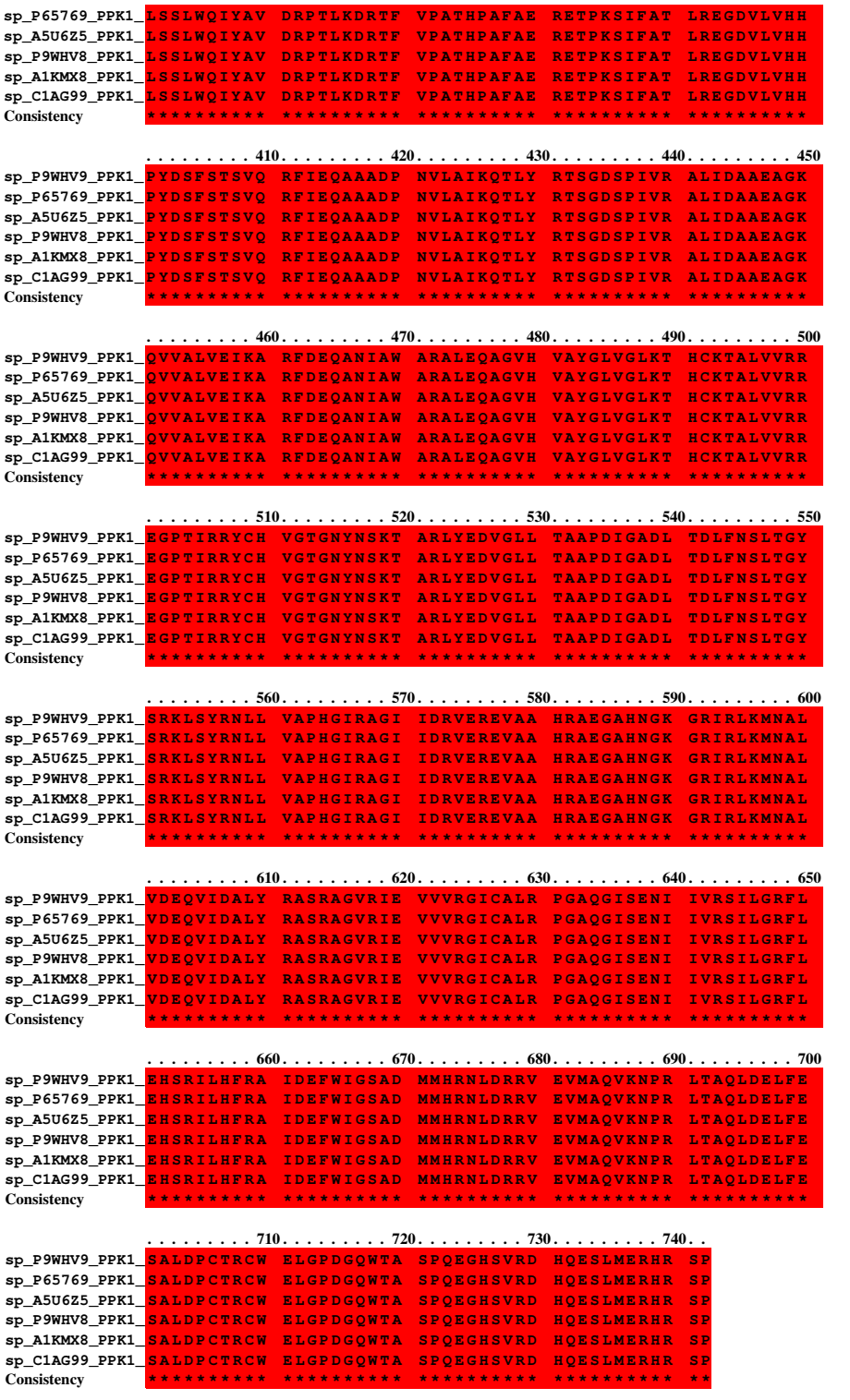

Supplement: Supplementary file 1 — Table S1 and Figure S1 [file 41598_2019_40621_MOESM1_ESM.docx]
